# Long-Term Outcome of Mechanical and Biological Prostheses in Patients with Left-Side Infective Endocarditis: A Systematic Review and Meta-Analysis

**DOI:** 10.3390/jcm10194356

**Published:** 2021-09-24

**Authors:** Francesco Formica, Francesco Maestri, Florida Gripshi, Alan Gallingani, Silvia Grossi, Francesco Nicolini

**Affiliations:** 1 Department of Medicine and Surgery, University of Parma, 43126 Parma, Italy; 2 Cardiac Surgery Unit, University Hospital of Parma, 43126 Parma, Italy; fmaestri@ao.pr.it (F.M.); florida.gripshi@gmail.com (F.G.); gallingalan@gmail.com (A.G.); 3 Department of Anesthesia and Intensive Care, Parma University Hospital, 43126 Parma, Italy; sigrossi@ao.pr.it

**Keywords:** infective endocarditis, mechanical prosthesis, biological prosthesis, meta-analysis, long-term outcome

## Abstract

Background. Long-term outcomes of patients with infective endocarditis (IE) who received either a mechanical (MP) or biological prosthesis (BP) are conflicting. A meta-analysis of observational studies comparing the long-term outcomes of left-side IE with the use of MP versus BP was performed. Methods. Electronic databases from January 2000 to June 2021 were screened. Studies reporting long-term mortality were analyzed. The primary endpoint was long-term overall mortality. Secondary endpoints were in-hospital/.30-day mortality and freedom from both prosthesis reinfection and reintervention. The pooled hazard ratio (HR) with 95% confidence interval (CI) was calculated for survival according to the random effect model. Results. Thirteen retrospective observational studies reporting on 8645 patients (MP: 4688; BP: 4137) were included for comparison. Twelve studies reported data of long-term survival for a total of 8285 patients (MP: 4517; BP: 3768). The pooled analysis revealed that the use of MP was statistically associated with longer benefits compared to BP (HR 0.74; 95% CI 0.63–0.86; *p* < 0.0001). The median follow-up time ranged from 1 to 15.3 years. The pooled analysis of five studies reporting data on prosthesis reinfection in 4491 patients (MP: 2433; BP: 2058) did not reveal significant differences (HR 0.60; 95% CI 0.30–1.21; *p* = 0.15). Five studies reported data on prosthesis reintervention in 4401 patients (MP: 2307; BP: 2094). The meta-analysis revealed a significant difference in favor of MP (HR 0.40; 95% CI 0.29–0.55; *p* < 0.0001). Meta-regression reported no effect of male gender (*p* = 0.09) and age (*p* = 0.77) on long-term survival. Conclusions. In a meta-analysis of retrospective observational studies comparing the long-term outcome of patients who underwent surgery for left-sided IE, the use of MP compared to BP is associated with a significant longer-term survival and with a reduced incidence of late reoperation. The incidence of late reinfection is comparable between the two prostheses.

## 1. Introduction

Infective endocarditis (IE) is a serious disease associated with a relevant in-hospital mortality ranging between 15% and 20% as well as a 1-year mortality approaching 40% [1]. Despite improvements in prevention, medical, and surgical management, the trend of IE has been continuously increasing over the last 20 years worldwide [2,3,4,5,6].

IE involves both native and prosthetic valves as well as prosthetic devices. Prosthetic valve endocarditis is associated with a higher incidence of annular abscess, extensive annular infection, and large tissue destruction, which can lead to higher in-hospital and long-term mortality compared to native valve endocarditis [7]. In patients with acute IE, the surgical treatment remains as the first option for many patients mainly in complex clinical scenarios characterized by persistent fever, extensive valvular tissue destruction with severe valve regurgitation, large perivalvular leakage, and acute cardiogenic shock. The most important part of the surgery, in combination with antimicrobic therapy, is the radical debridement of all infected and necrotic tissue, and the removal of all prosthetic material to obtain a new anatomic condition in which the microcolony of microbes cannot produce a biofilm matrix, which is a self-produced matrix of extracellular polymeric substances that cause persistent inflammation, systemic embolization, and new biofilm formation [8]. When surgery is indicated, valve repair is preferred over valve replacement, especially in mitral valve endocarditis. However, most of the left side endocarditis is managed with valve replacement due to extensive tissue destruction. Currently, there are conflicting results related to the type of prosthesis to implant, i.e., whether it should be a mechanical or biological valve. Moreover, the most recent guidelines on IE [9,10] do not support the superiority of one prosthesis over the other one and no randomized clinical trials have been performed. Some reports have compared the early and long-term outcomes of patients who received a mechanical or a biological prosthesis; however, the results are conflicting and controversial. To overcome this limitation, we have undertaken this meta-analysis with available evidence to compare the long-term outcomes of left-sided endocarditis in patients who received mechanical prosthesis (MP) compared to biological prosthesis (BP).

## 2. Materials and Methods

### 2.1. Search Strategy and Study Selection

The systematic review and meta-analysis were performed according to the Preferred Reporting Items for Systematic Reviews and Meta-Analyses (PRISMA) guidelines [11]. The study protocol was registered and published online in PROSPERO (The International Prospective Register of Systematic Reviews; ID: CRD42021246445).

We performed a systematic search from 1st January 2000 to 30th June 2021 in three electronic databases: PubMed, EMBASE, and SCOPUS. Search terms used alone or in combination included “infective endocarditis” AND “mechanical prosthesis” OR “biological prosthesis” OR “bioprosthesis” OR “tissue valve” OR “bioprosthetic valve” OR “long-term results”.

This systematic review answered targeted questions that aimed to solve a clinical problem following the PICOS format (population; intervention; comparison; outcomes; and studies).

Population: patients with infective endocarditis of native or prosthetic valve. Intervention: replacement of infected native or prosthetic valve with prosthesis. Comparison: mechanical valve versus bioprosthetic valve. Outcomes: long-term survival and freedom from both reintervention and reinfection. Studies: retrospective observational studies, propensity score-matched studies, and randomized controlled trials.

Relevant studies were selected if they met the following criteria: (1) patients who have undergone left heart valve surgical replacement; (2) patients who have received either a mechanical or biological prosthesis; (3) long-term survival, comparing patients with a mechanical prosthesis to patients with a biological prosthesis; (4) freedom from reintervention and reinfection, comparing patients with a mechanical prosthesis to patients with a biological prosthesis; and (5) evidence of Kaplan–Meier curve for survival analysis.

Only studies written in English languages were included. Editorials, letters, case reports, meta-analyses, and review articles were excluded from the analysis. Studies with incomplete or inadequate data mainly regarding the long-term survival, studies that reported survival of less than 12 months, and studies with a number of patients of less than 15 for each group were not considered for the analysis.

Two authors (FM and FG) independently selected and reviewed both titles and abstracts to identify articles meeting the inclusion criteria. Any disagreement was reviewed by a third senior author (FF) who made the decision of whether to include or exclude the study.

### 2.2. Data Collection and Extraction

Data were extracted independently by two (FM and FG) authors and the total number of events was reported in a standard table sheet (Microsoft Office Excel 2016, Microsoft, Redmond, WA, USA). Kaplan–Meier curve graphs were digitalized using GetData Graph Digitizer version 2.26.0.20 (http://getdata-praph-digitizer.com (accessed on 15 April 2021)) and finally both the hazard ratio (HR) and the corresponding 95% confidence interval (CI) were calculated [12,13]. 

Median and interquartile ranges were converted into mean and standard deviations as suggested by Luo et al. [14].

Each study included in the meta-analysis was identified by study design, first author, country, and year of publication.

The following patient preoperative variables were recorded: age, male gender, diabetes, renal failure, acute infective endocarditis, acute heart failure, stroke, and septic shock.

### 2.3. Endpoints

The primary endpoint was long-term mortality. For this endpoint, HR and the corresponding 95% CI were extracted, were compared with the original HR if reported, and were considered in the analysis. The secondary endpoints were: (i) early mortality, which was defined as mortality occurring within 30 days or during the index hospitalization; (ii) freedom from prosthesis reinfection; and (iii) freedom from prosthesis reintervention.

### 2.4. Statistical Analysis

The pooled hazard ratio (HR) and 95% CI by the Mantzel–Haenszel method were calculated for survival analysis and for freedom from both prosthesis reinfection and reintervention. The pooled odd ratio (OR) and 95% CI by the Mantzel–Haenszel method were calculated for 30-day/in-hospital mortality. Mechanical prosthesis was the reference group. The random effect model was chosen due to the wide heterogeneity of patient populations. Forest plots were created to visualize the outcomes and to calculate the effect size. Statistical heterogeneity was assessed with the chi-square test and I^2^ test. Heterogeneity was defined as low when I^2^ ranged from 0 to 25%, moderate when I^2^ ranged from 26 to 50%, and high when I^2^ was above 50% [15]. 

For the survival analysis, we reconstructed the database of each article using the method described by Wei et al. [16]. The obtained survival curves were then visually compared with the original curves. All data were pooled into one global database and life tables, survival curves, and HR were calculated. The log-rank test was used to determine differences between the two groups. A stratified Cox proportional hazard model was used to calculate the HR of MP versus BP.

The publication bias was graphically represented with a funnel plot using the trim and fill method, and was analyzed using both Egger’s test [17] and Begg and Mazumdar’s test [18]. Sensitivity analysis was used to verify the influence of a single study on the overall effect of MP on the main outcome, following the leave-one-out method [19]. Meta-regression analysis was run to determine if the effects of MP were modulated by pre-identified factors. The factors used in the meta-regression were male gender and age.

Mean age was weighted by the number of patients in the included studies. Continuous variables were reported as mean ± standard deviation. Categorical variables were reported as percentages. A two-tailed *p*-value < 0.05 was considered statistically significant. All the statistical analyses were computed and analyzed with ProMeta3 software (http://idostatistics.com/prometa3/ (accessed on 15 Jannuary 2021)) and Stata/SE 16.1 (Lakeway, TX, USA).

## 3. Results

### 3.1. Study Selection and Literature Search

**Figure 1:**
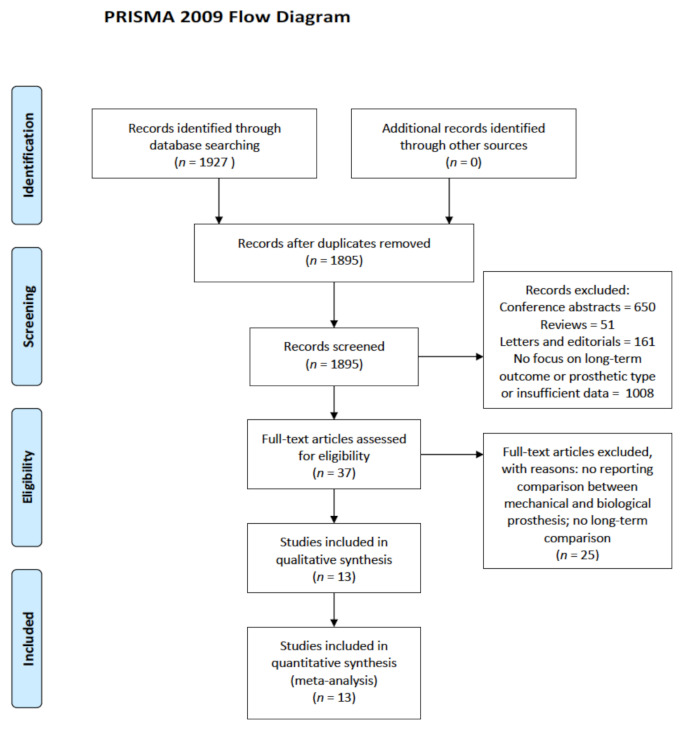
Study selection according to the Preferred Reporting Items for Systematic Reviews and Meta-Analyses (PRISMA), presented as a flow diagram.

The search strategy identified a total of 1927 titles and abstracts, of which 37 full-text articles were evaluated for the meta-analysis. After the review of full-text articles, 13 retrospective observational studies [19,20,21,22,23,24,25,26,27,28,29,30,31] met our eligibility criteria and were included in this meta-analysis. A PRISMA flow chart of the study selection process is shown in Figure 1.

Patient characteristics and the study typology are reported in Table 1 and Table 2. Overall, the included studies reported on a total of 8645 patients with infective endocarditis, including 4688 patients in the MP group and 4137 in BP group.

The Risk of Bias in Non-randomized Studies of Interventions tool (ROBINS-I) for non-randomized observational studies confirmed the level of quality of the selected articles (Supplementary Figure S1).

### 3.2. Study Primary Endpoint: Long-Term Mortality

**Figure 2:**
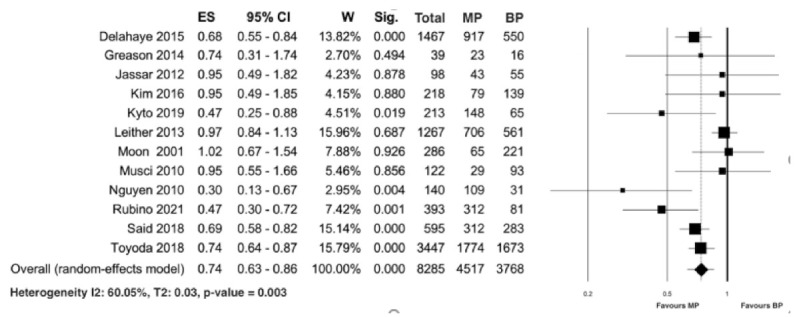
Forest plot for long-term survival. Abbreviations: ES, effect size, indicating the hazard ratio; CI, confidence interval; W, weight; MP, mechanical prosthesis; and BP, biological prosthesis.

The overall weighted mean age of patients who underwent surgery for infective endocarditis was 55.1 years (95% CI, 52.3–60.2) and was calculated from 11 studies reporting the mean age of all patients [19,20,21,22,23,24,26,27,28,29,30]. Eight studies [21,22,24,27,28,30,31,32] reported the mean age of patients receiving either a MP or BP. In the mechanical group, the weighted mean age was 49.2 years (CI, 47.1–55.3), and in the biological group, the weighted mean age was 59.8 years (CI, 55.8–63.2). Twelve studies [20,21,23,24,25,26,27,28,29,30,31,32] reporting on 8285 patients with infective endocarditis, including 4517 patients with MP and 3768 with BP, analyzed the overall long-term comparison between MP and BP, with a median follow-up ranging from 1 to 15.3 years (20,27). The longest follow-up was 20 years [20]. The pooled analysis of long-term survival revealed a significant difference between the two groups, favoring MP (random effect model: HR 0.74; 95% CI 0.63–0.86; *p* < 0.0001), with evidence of significative heterogeneity (I2: 60.05%; T2: 0.03; *p*-value = 0.003; Figure 2).

**Figure 3:**
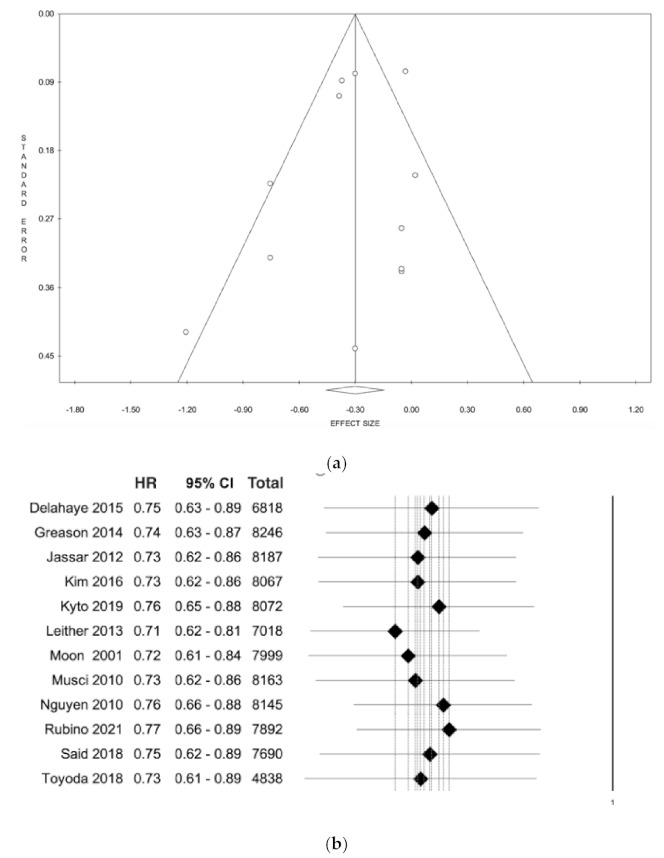
(**a**) Funnel plot to assess the publication bias. No publication bias was reported relating to the long-term survival. Circles define the position of each study in the funnel plot (**b**) Forest plot of the leave-one-out analysis comparing the effect of the use of a mechanical and biological prosthesis. Abbreviation: HR, hazard ratio.

The funnel plot analysis did not show asymmetry for the outcomes, suggesting an absence of publication bias (Egger’s linear regression test: *p* = 0.39; Begg and Mazumdar’s test: *p* = 0.27; Figure 3a). The leave-one-out analysis did not reveal any significant impact on long-term survival with any one study being removed (Figure 3b).

**Figure 4:**
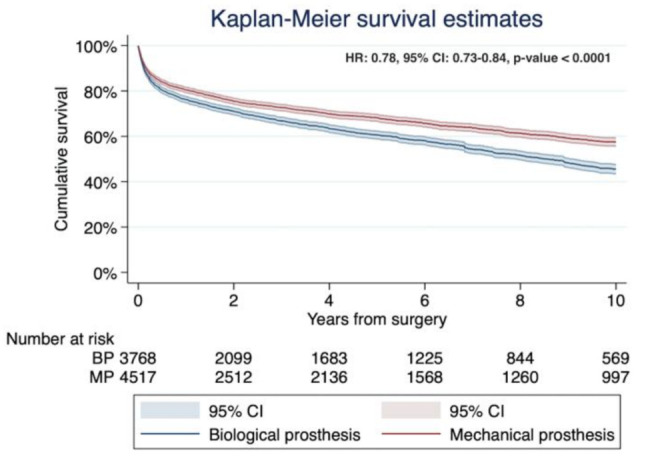
Kaplan–Meier survival curves of the pooled sample. The MP group was associated with better long-term survival compared to the BP group. All databases were merged into one global database. The log-rank test was statistically significant, favoring the MP group (*p* < 0.0001). Abbreviations: HR, hazard ratio; CI, confidence interval; MP, mechanical prosthesis; and BP, biological prosthesis.

The pooled Kaplan–Meier curve is shown in Figure 4. The long-rank test was statistically significant, favoring the mechanical prosthesis (*p* < 0.0001). The survival rate for the mechanical prosthesis versus biological prosthesis at 2, 4, 6, and 10 years were: 72.7%, 68.2%, 63.8%, and 56.5%, and 66.7%, 60.6%, 54.3%, and 43%, respectively. These indicate the numbers needed to treat 9.1 patients at a 10-year follow-up. The Cox regression indicated that the risk of death was significatively reduced by 22% in patients with MP compared to patients in the BP group (HR 0.78; CI 0.73–0.84; *p* < 0.0001).

**Figure 5:**
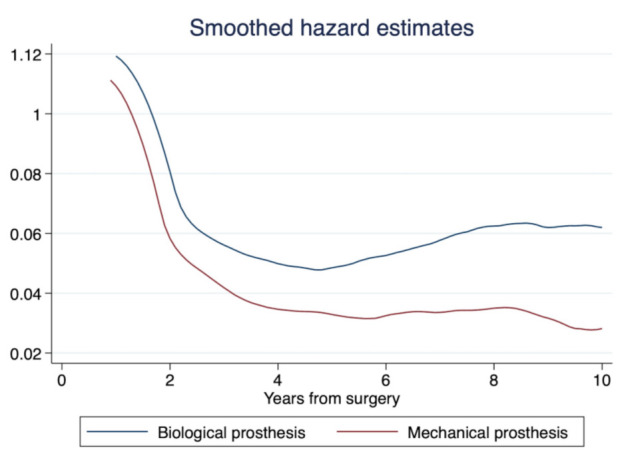
Smoothed hazard function of the all databases pooled in one global database.

A smoothed hazard function according to the type of treatment is shown in Figure 5.

**Figure 6:**
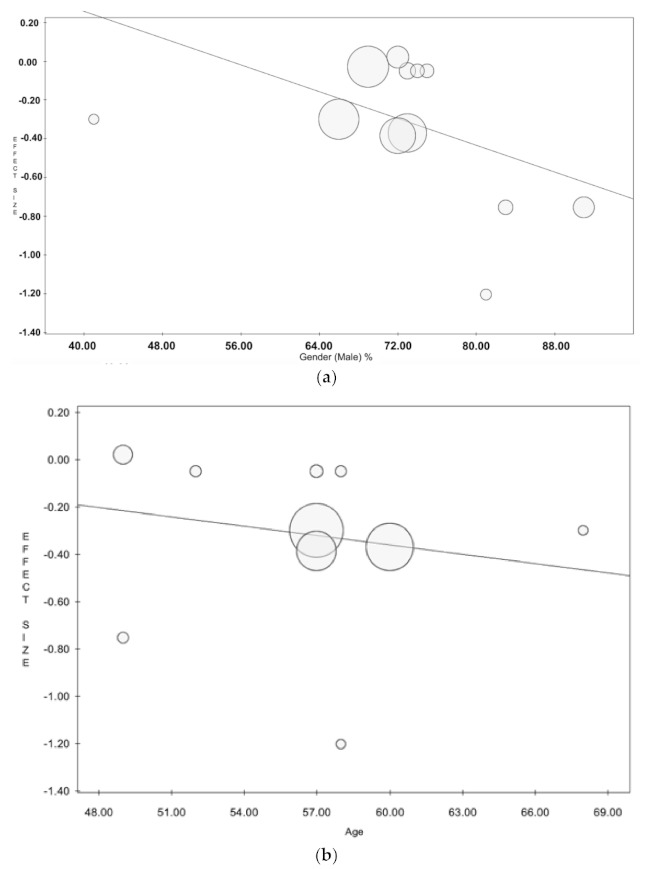
(**a**) Meta-regression analysis plot: effect of male gender. No effect of male gender was reported on long-term survival (*p* = 0.092). (**b**) Meta-regression analysis plot: effect of age. No effect of age was reported on long-term survival (*p* = 0.776).

As shown in Figure 6, the meta-regression analysis revealed no effect of male gender (*p* = 0.092) and age (*p* = 0.776). Unfortunately, there was no sufficient data to perform a meta-regression to adjust for diabetes, renal failure, acute infective endocarditis, acute heart failure, stroke, and septic shock.

### 3.3. Study Secondary Outcome: Early Mortality and Freedom from Both Prosthesis Reinfection and Reintervention

**Figure 7:**
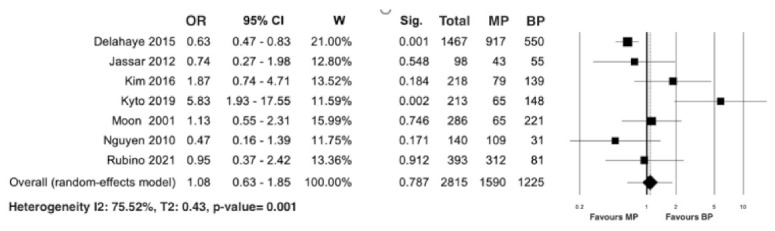
Forest plot for early mortality. No statistical differences were observed between the MP and BP groups. Abbreviations: OR, odd ratio; CI, confidence interval; W, weight; MP, mechanical prosthesis; and BP, biological prosthesis.

Seven studies presented data on early mortality [20,21,24,27,28,31,32] in 2815 patients, including 1590 with MP and 1225 with BP. The meta-analysis of the calculated OR did not reveal significant differences between the two groups (HR 1.08; 95% CI 0.63–1.85; *p* = 0.78). There was a significant heterogeneity (I^2^ = 75.52%; T2 = 0.43; *p* = 0.001; Figure 7). The leave-one-out analysis did not reveal any significant impact on early mortality with any one study being removed.

**Figure 8:**
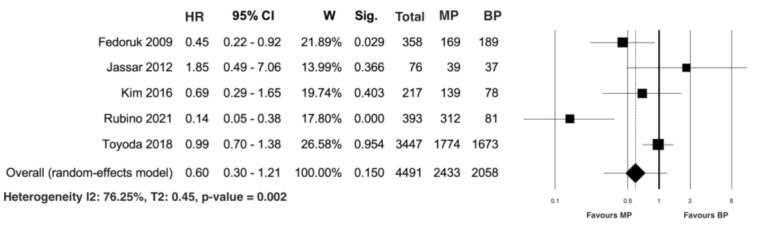
Forest plot for prosthesis reinfection. No statistical differences were observed between the MP and BP groups. Abbreviations: HR, hazard ratio; CI, confidence interval; W, weight; MP, mechanical prosthesis; and BP, biological prosthesis.

Five studies [22,24,28,30,32] reported data on late prosthesis reinfection in 4491 patients, including 2433 in the mechanical group and 2058 in the biological group. The pooled analysis of the calculated HR did not reveal significant differences between the patients treated with a MP compared to those treated with a BP (HR 0.60; 95% CI 0.30–1.21; *p* = 0.15). Heterogeneity was significant (I^2^ = 76.25%; T2 = 0.45; *p* value = 0.002; Figure 8). The leave-one-out analysis did not reveal any significant impact on late prosthesis reinfection with any one study being removed.

**Figure 9:**
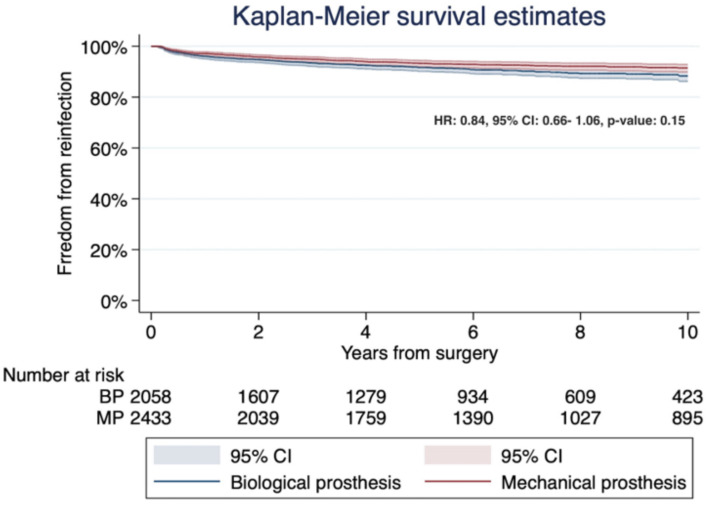
Kaplan–Meier curves detailing the freedom from prosthesis reinfection. No statistical differences were reported between mechanical and biological prostheses. Abbreviations: HR, hazard ratio; CI, confidence interval; MP, mechanical prosthesis; and BP, biological prosthesis.

The pooled Kaplan–Meier curve is shown in Figure 9. The long-rank test was not statistically significant (*p* = 0.13). Freedom from prosthesis reinfection for MP versus BP at 2, 4, 6, and 10 years were: 95.5%, 93.5%, 92.5%, and 91.1%, and 94.4%, 92.3%, 91.1%, and 89.3%, respectively. These indicate the number needed to treat 55.6 patients at the 10-year follow-up. The HR was 0.84 (95% CI: 0.66–1.06; *p* = 0.15). The funnel plot analysis did not demonstrate a publication bias (Egger’s linear regression test: *p* = 0.41; Begg and Mazumdar’s test: *p* = 0.62).

**Figure 10:**
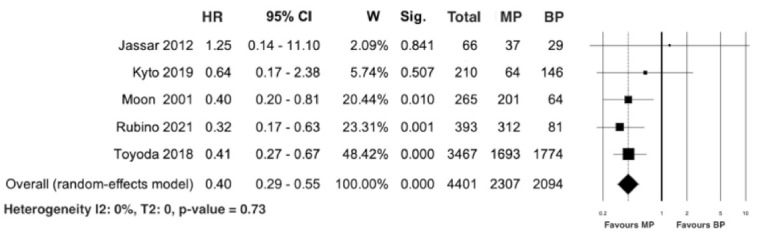
Forest plot for prosthesis reintervention. Abbreviations: HR, hazard ratio; CI, confidence interval; W, weight; MP, mechanical prosthesis; and BP, biological prosthesis.

Five studies [20,24,30,31,32] reported data on prosthesis reintervention in 4401 patients, including 2307 in the mechanical group and 2094 in the biological group. The meta-analysis of the calculated HR revealed a significant difference in favor of patients with MP compared to those treated with BP (HR 0.40; 95% CI 0.29–0.55; *p* < 0.0001), with the absence of heterogeneity (I2 = 0%; T2 = 0; *p* = 0.73; Figure 10). The leave-one-out analysis did not demonstrate any significant impact on prosthesis reintervention with any one study being removed.

**Figure 11:**
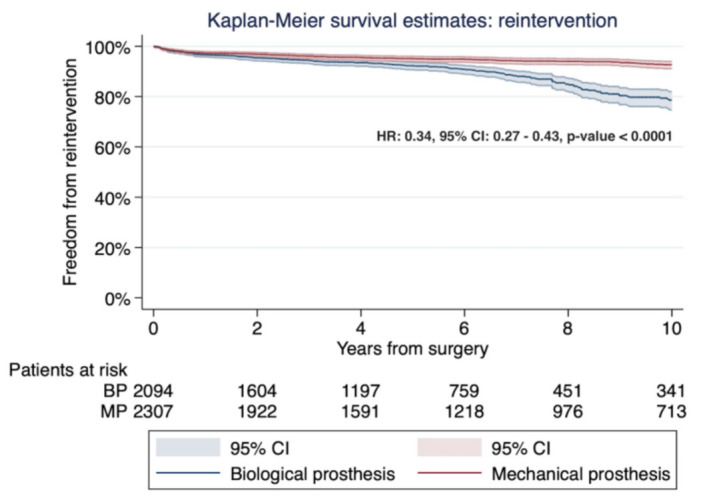
Kaplan–Meier curves detailing the freedom from prosthesis reintervention. The MP group is associated with a lower risk of reintervention compared to the BP group. The log-rank test was statistically significant, favoring the MP group (*p* < 0.0001). Abbreviations: HR, hazard ratio; CI, confidence interval; MP, mechanical prosthesis; and BP, biological prosthesis.

The pooled Kaplan–Meier curve is shown in Figure 11. The long-rank test was statistically significant, favoring the mechanical prosthesis (*p* < 0.0001). Freedom from prosthesis reintervention for the mechanical prosthesis versus biological prosthesis at 2, 4, 6, and 10 years were: 96.8%, 95.5%, 94.8%, and 92.6%, and 95.4%, 93.5%, 90.8%, and 78.7%, respectively. These indicate the number needed to treat 7.2 patients at the 10-year follow-up. The Cox regression indicated that the risk of reintervention was reduced by 66% in patients with a mechanical prosthesis compared to patients with a bioprosthetic valve (HR: 0.34; 95% CI: 0.27–0.43; *p* < 0.0001). The funnel plot analysis did not reveal a publication bias (Egger’s linear regression test: *p* = 0.14; Begg and Mazumdar’s test: *p* = 0.14).

## 4. Discussion

The major findings of the meta-analysis were that in patients who underwent surgery for left-sided endocarditis, (i) the mechanical prosthesis was associated with longer-term survival compared to a biological prosthesis; (ii) the incidence of late prosthesis reinfection was comparable between the two prostheses; and (iii) the mechanical prosthesis was associated with a lower probability of late reoperation compared to a biological valve. The meta-regression analysis revealed that age and male gender did not affect the long-term survival. Unfortunately, there were no sufficient data to perform a reliable meta-regression analysis to adjust for other preoperative conditions such as active infective endocarditis, cardiogenic shock, renal failure, hemorrhagic and thrombotic cerebral events, and septic shock.

We performed, to the best of our knowledge, the largest meta-analysis to date examining the studies published during the last two decades. Recently, two meta-analyses reported conflicting results. Tao et al. [33] performed a meta-analysis of 11 studies published from 1960 to 2016. Among them, eight studies were published from 2000 and after. The authors selected 10,754 patients, including 6776 in the biological group and 3978 in the mechanical group. They reported a significantly higher risk of death for all-cause mortality in the biological prosthesis group than in the mechanical group (HR 1.22; 95% CI 1.003–1.44). Recurrence of endocarditis, which was reported in five studies, was statistically higher in patients with BP (HR 1.75; 95% CI 1.26–2.42). The incidence of reoperation (which was reported in six studies) was statistically higher in the BP group (HR 1.79; 95% CI 1.15–2.80). No statistical difference related to post-operative embolic events was observed between the two prostheses. This data was reported in only four studies. A more recent meta-analysis covered 11 studies, including 2057 patients receiving a BP and 2336 patients receiving a MP [34]. Long-term survival data were reported in ten studies. The authors observed a comparable 10-year survival between biological and mechanical valves (HR 0.94; 95% CI 0.73–1.21). Composite 10-year survival was 37.5% for biological prostheses and 34% for mechanical valves. The authors did not observe statistical differences in terms of prosthesis reinfection (data extrapolated from three studies) and reoperation (data extrapolated from four studies). However, the authors acknowledged the difficulty of performing a robust analysis due to the complexity of the course of IE and the multiple factors that lead to prosthesis-related decision-making, and therefore did not recommend or suggest any strategy in terms of prosthesis selection.

The process for prosthesis selection for the general population is well defined in the most recent North American and European guidelines [35,36]. North American guidelines recommend the use of a mechanical valve to replace the aortic valve in patients younger than 50 years old and a mitral valve replacement for those younger than 65, unless there is a contraindication for long-life anticoagulation therapy (Class II-A), while bioprostheses are recommended for patients over 65 years old (Class II-A). For the 50–65-years-old window and for patients needing aortic valve replacement, it is reasonable to individualize the choice of the prosthesis by considering the patient’s risk factors, personal wishes, clinical conditions, and life expectancy (Class II-A) [35]. The European guidelines recommend a mechanical prosthesis in the aortic position for patients <60 years and in the mitral position for patients <65 years (Class II-A). In contrast, a bioprosthesis is recommended in situations including the following factors: patient’s personal wish; poor long-term anticoagulation control; high risk of bleeding (Class I-A); low risk of future redo valve surgery; women contemplating pregnancy; age over 65 years in the aortic position; >70 years old in the mitral position; and a life expectancy lower than the predicted durability of the biological prosthesis (Class II-A) [36]. The prosthesis selection for patients with IE remains more complex than for the population without endocarditis. Usually, for patients with a high risk of intracranial bleeding, the bioprosthesis has to be considered as the first choice regardless of the patient’s age (Class II-A) [10].

During the last decades, the scientific community has been continuously faced with progressive changes in heart valve management. The results of valve-in-valve therapy [37] are promising and encouraging. This is in favor of the increased use of biological prostheses even in patients under 60 years of age. In addition, the latest generation of biological prostheses with novel preservation technology to protect against valve degeneration [38] and other prosthesis predisposed to future valve-in-valve therapy [39] are being used in clinical practice with the aim of eliminating long-life anticoagulant therapy. In addition, some last generation mechanical prostheses with lower thromboembolism incidence would allow for less anticoagulation and therefore have a wider use in patients >60 years old [40]. In analyzing the impact of both mechanical and biological prostheses on long-term survival, late prosthesis reinfection and late reoperation are crucial elements in the prosthetic type selection for a complex clinical scenario such as infective endocarditis. We therefore preferred to focus on long-term results rather than intra-hospital outcome. In these contexts, we observed a clear association of longer-term benefits at 10 years with a mechanical prosthesis compared to a bioprosthesis. These data are derived from the pooled data of 12 studies and we believe this is a major strength of the meta-analysis. On the contrary, data related to reinfection and reintervention are derived from five studies, hence reducing the robustness and interpretation of the results. However, considering the comparable incidence of late reinfection for both prostheses as well as the higher incidence of late reintervention for the biological prosthesis compared to the mechanical prosthesis, we can speculate that IE per se may not be relevant for the durability of the prostheses and therefore the choice of the prosthetic valve should be guided by the current guidelines unless a complex clinical scenario dictates individualization of the prosthesis choice. We have observed that the curves of long-term survival begin to diverge at about 1 year and at 3 years, the two curves diverge significatively; this similar trend was observed between the survival curves, showing the incidence of reoperation, at which the curves start to diverge at about 6 years. We have no data related to the criteria used by the authors to select the type of prosthesis and therefore we cannot give any robust results. We can speculate that in each selected study, the authors mainly adopted the age cut-off criteria for prosthesis selection, therefore implanting a mechanical prosthesis in younger patients. This may explain the reason why the mechanical prosthesis was associated with longer-term survival. Moreover, we can also hypothesize that the better benefit associated with the mechanical prosthesis is in part related to the lower incidence of late reoperation. Unfortunately, only five studies reported the rate of reoperation and therefore it was not possible to perform an adequate analysis to verify a direct correlation between overall long-term survival and freedom from reoperation. We were able to perform a meta-regression analysis to correct the long-term overall survival for age because this variable was reported in 11 studies. Meta-regression revealed that age at the time of surgery had no effect on long-term overall survival. It is important to emphasize that the difference in mean age between the two groups is relevant. The weighted mean age in patients with MP is 49.2 and in patients with BP is 59.8. Unfortunately, only seven studies [21,22,27,28,30,31,32] of the twelve included the mean age data for each group in the survival analysis reported. Therefore, because of a lack of consistency in the presentation of the data and results of the studies included in the meta-analysis, an appropriate statistical analysis to determine the impact of the age factor on long-term survival was not possible. Although some authors have reported increasing age as an independent predictor of late death risk [20,21,27,29], other authors [24,30,31] failed to demonstrate an independent correlation between age and late survival. Therefore, considering that the cumulative overall survival of the studies included in the meta-analysis shows that the mechanical valve is associated with longer-term benefits and that age at the time of surgery had no effect on late survival, we can assume that the use of a mechanical prosthesis is safe in patients with infective endocarditis and can be implanted regardless of the patient’s age. Additionally, we can assume it is better, instead, to consider the patient’s clinical status and desire. It is worthy to note that the incidence of late reinfection was comparable between the two types of prostheses and this result may support our hypothesis.

### Limitations

This study has limitations regarding the fact that meta-analyses of retrospective observational studies can be affected by a substantial risk of treatment allocation bias. The surgeon’s decision to use either the mechanical or biological prosthesis cannot be eliminated by any statistical method. Moreover, unmeasured or unknown confounders cannot be controlled for by meta-analysis studies, resulting in potential bias.

Only twelve observational studies were analyzed for the meta-regression, which is perhaps a limitation of the statistical significance and interpretation of the meta-regression considering only age at the time of surgery and gender were reported in all studies. For primary research, it is strongly advised to apply the rule of 10 subjects for each covariate to perform a robust linear regression; this would correspond to 10 studies for each moderator in the meta-regression analysis [41].

Additionally, some of the studies included in this analysis are limited by a relatively small number of patients and this may emphasize the selection bias, which is inherent in each study as highlighted by the evidence of significant heterogeneity. Further data, however, are still required.

## 5. Conclusions

In conclusion, in a meta-analysis of retrospective observational studies comparing the long-term outcomes of patients who underwent surgery for left-side infective endocarditis, the use of mechanical prosthesis was associated with a significant survival benefit at long-term follow-up as well as with a reduced incidence of late reoperation. The incidence of late reinfection was comparable between the two prostheses. These findings may encourage an even wider use of mechanical prostheses in cases of infective endocarditis and can limit the reasonable doubts that many surgeons have at the time of surgery. Obviously, recent guidelines, clinical factors, patient age, particular anatomic conditions, patient’s personal wishes, and the surgeon’s decision always remain as indispensable cornerstones to be considered in the prosthetic choice. Further prospective multicenter studies and randomized trials are needed to better define the most appropriate prosthesis to use in infective endocarditis.

## Figures and Tables

**Table 1:** Study typology and patients’ characteristics: (**a**) total patients do not include 20 homografts; (**b**) total patients do not include 27 homografts; (**c**) total patients do not include 103 homografts; (**d**) total patients do not include 36 homografts; (**e**) total patients do not include 86 homografts; and (**f**) total patients do not include 283 homografts and 122 valve repairs. Abbreviations: MP, mechanical prosthesis; BP, biological prosthesis; ROS, retrospective observational study; and n.a., not available.

Authors (Years/Country/Study Design)	Number of Patients (%)	Male Gender (%)	Mean Age	DM (%)	RF (%)
**^a^** Moon et al. [20] (2001/USA/ROS)	Total 286	Total: 221 (72)	Total: 49	Total: 32 (10.4)	Total: 78 (25.5)
	MP: 65 (22.7)	MP: n.a.	MP: n.a.	MP: n.a.	MP: n.a.
	BP: 221 (77.3)	BP: n.a.	BP: n.a.	BP: n.a.	BP: n.a.
**^b^** Nguyen et al. [21] (2009/France/ROS)	Total: 140	Total: 113 (80.7)	Total: 57.9	Total: 17 (12.1)	Total: 15 (10.7)
	MP: 109 (77.8)	MP: 88 (80.7)	MP: 57.3	MP: 14 (12.8)	MP: 11 (10.1)
	BP: 31 (22.2)	BP: 25 (80.6)	BP: 63.2	BP: 3 (9.7)	BP: 4 (12.9)
Fedoruk et al. [22] (2009/Canada/ROS)	Total: 358	Total: 257 (71.8)	Total: 41.8	Total: n.a.	Total: n.a.
	MP: 169 (47.2)	MP: n.a.	MP: 45.6	MP: n.a.	MP: n.a.
	BP: 189 (52.8)	BP: n.a.	BP: 51.6	BP: n.a.	BP: n.a.
**^c^** Musci et al. [23] (2010/Germany/ROS)	Total 122	Total: 255 (73.1)	Total: 57.3	Total: 76 (21.8)	Total: 130 (37)
	MP: 29 (23.8)	MP: n.a.	MP: n.a.	MP: n.a.	MP: n.a.
	BP: 93 (76.2)	BP: n.a.	BP: n.a.	BP: n.a.	BP: n.a.
**^d^** Jassar et al. [24] (2012/USA/ROS)	Total: 98	Total: 73 (74.5)	Total: 58.3	Total: 17 (17.3)	Total: 13 (13.3)
	MP: 43 (43.9)	MP: 38 (88.3)	MP: 51.8	MP: 9 (20.9)	MP: 3 (7)
	BP: 55 (56.1)	BP: 35 (63.6)	BP: 62.7	BP: 10 (18.2)	BP: 10 (18.2)
Leither et al. [25] (2013/USA/ROS)	Total: 1267	Total: 763 (69.2)	Total: n.a.	Total: 454 (35.8)	Total: 1267 (100)
	MP: 761 (60)	MP: 417 (61.7)	MP: n.a.	MP: 241 (34.1)	MP: 761 (100)
	BP: 506 (40)	BP: 346 (59.1)	BP: n.a.	BP: 212 (37.8	BP: 506 (100)
Greason et al. [26] (2014/USA/ROS)	Total: 39	Total: 16 (41)	Total: 68	Total: n.a.	Total: 6 (15)
	MP: 23 (59)	MP: n.a.	MP: n.a.	MP: n.a.	MP: n.a.
	BP: 16 (41)	BP: n.a.	BP: n.a.	BP: n.a.	BP: n.a.
Delahaye et al. [27] (2014/Multicenter/ROS)	Total: 1467	Total: 1053 (71.9)	Total: 56.6	Total: n.a.	Total: 88 (12.6)
	MP: 917 (62.5)	MP: 665 (72.7)	MP: 53.6	MP: n.a.	MP: 39 (9.5)
	BP: 550 (37.5)	BP: 388 (70.5)	BP: 61.6	BP: n.a.	BP: 49 (17.1)
**^e^** Kim et al. [28] (2016/South Korea/ROS)	Total: 218	Total: 165 (75.8)	Total: 52.3	Total: 36 (16.5)	Total: n.a.
	MP: 79 (36.2)	MP: 60 (75.5)	MP: 47.2	MP: 6 (5.6)	MP: n.a.
	BP: 139 (63.8)	BP: 105 (75.9)	BP: 59.8	BP: 30 (21.6)	BP: n.a.
**^f^** Said et al. [29] (2017/USA(ROS)	Total: 595	Total: n.a.	Total: n.a.	Total: n.a.	Total: n.a.
	MP: 312 (52.4)	MP: n.a	MP: n.a	MP: n.a	MP: n.a
	BP: 283 (47.6)	BP: n.a	BP: n.a	BP: n.a	BP: n.a
Toyoda et al. [30] (2018/USA/ROS)	Total: 3247	Total: 2295 (70.7)	Total: n.a.	Total: 332 (10.2)	Total: 738 (22.7)
	MP: 1574 (48.5)	MP: 1207 (70.7)	MP: 53.4	MP: 163 (10.3)	MP: 365 (23.2)
	BP: 1673 (51.5)	BP: 1086 (64.9)	BP: 60.4	BP: 169 (10.1)	BP: 373 (22.3)
Kyto et al. [31] (2019/Finland/ROS)	Total: 213	Total: 177 (83.1)	Total: 49.1	Total: n.a.	Total: n.a.
	MP: 148 (69.5)	MP: 130 (87.8)	MP: 47.1	MP: n.a.	MP: n.a.
	BP: 65 (30.5)	BP: 47 (72.3)	BP: 53.7	BP: n.a.	BP: n.a.
Rubino et al. [32] (2021/Italy/ROS)	Total: 395	Total: 296 (74.9)	Total: n.a.	Total: 46 (11.6)	Total: 21 (5.3)
	MP: 314	MP: 235 (74.8)	MP: 50.1	MP: 39 (12.4)	MP: 19 (6.1)
	BP: 81	BP: 61/75.3)	BP: 56.3	BP: 7 (8.6)	BP: 2(2.5)

**Table 2:** Study typology and patients’ characteristics.

Authors (Years/Country)	Active Endocarditis (%)	Cardiogenic Shock (%)	Stroke (%)	Septic Shock (%)
Moon et al. [20] (2001/USA/ROS)	Total: 211 (68.9)	Totale: 218 (71.2)	n.a.	Total: 20 (6.5)
	MP: n.a.	MP: n.a.	MP: n.a.	MP: n.a.
	BP: n.a.	BP: n.a.	BP: n.a.	BP: n.a.
Nguyen et al. [21] (2009/France/ROS)	Total: 140 (100)	Total: 38 (27.1)	Total: 20 (14.2)	Total: 13 (9.3)
	MP: 109 (100)	MP: 31 (28.4)	MP: 19 (17.4)	MP: 9 (8.2)
	BP: 31 (100)	BP: 7 (22.6)	BP: 1 (14.3)	BP: 4 (12.9)
Fedoruk et al. [22] (2009/Canada/ROS)	Total: n.a.	Total: n.a.	Total: n.a.	Total: n.a.
	MP: n.a.	MP: n.a.	MP: n.a.	MP: n.a.
	BP: n.a.	BP: n.a.	BP: n.a.	BP: n.a.
Musci et al. [23] (2010/Germany/ROS)	Total: 42 (34.4)	Total: 35 (10)	Total: 25 (7.2)	Total: 37 (10.6)
	MP: 9 (21.42)	MP: n.a.	MP: n.a.	MP: n.a.
	BP: 33 (78.6)	BP: n.a.	BP: n.a.	BP: n.a.
Jassar et al. [24] (2012/USA/ROS)	Total: 98 (100)	Total: 7 (7.1)	Total: 30 (30.6)	Total: n.a.
	MP: 43 (100)	MP: 3 (3)	MP: 13 (30.2)	MP: n.a.
	BP: 55 (100)	BP: 4 (11.3)	BP: 17 (30.9)	BP: n.a.
Leither et al. [25] (2013/USA/ROS)	Total: n.a.	Total: n.a.	Total: 399 (31.5)	Total: n.a.
	MP: n.a.	MP: n.a.	MP: 278 (36.6)	MP: n.a.
	BP: n.a.	BP: n.a.	BP: 186 (36.8)	BP: n.a.
Greason et al. [26] (2014/USA/ROS)	Total: 12 (31)	Total: 22 (56)	Total: n.a.	Total: 6 (15)
	MP: n.a.	MP: n.a.	MP: n.a.	MP: n.a.
	BP: n.a.	BP: n.a.	BP: n.a.	BP: n.a.
Delahaye et al. [27] (2014/Multicenter/ROS)	Total: 1467 (100)	Total: n.a.	Total: n.a.	Total: n.a.
	MP: 917 (100)	MP: n.a.	MP: n.a.	MP: n.a.
	BP: 550 (100)	BP: n.a.	BP: n.a.	BP: n.a.
Kim et al. [28] (2016/South Korea/ROS)	Total: 218 (100)	Total: 10 (4.6)	Total: n.a.	Total: n.a.
	MP: 79 (100)	MP: 5 (6.3)	MP: n.a.	MP: n.a.
	BP: 139 (100)	BP: 139 (3.6)	BP: n.a.	BP: n.a.
Said et al. [29] (2017/USA/ROS)	Total: n.a.	Total: n.a.	Total: n.a.	Total: n.a.
	MP: n.a.	MP: n.a.	MP: n.a.	MP: n.a.
	BP: n.a.	BP: n.a.	BP: n.a.	BP: n.a.
Toyoda et al. [30] (2018/USA/ROS)	Total: 3247 (100	Total: 1974 (60.8)	Total: n.a.	Total: n.a.
	MP: 1574 (100)	MP: 981 (62.3)	MP: n.a.	MP: n.a.
	BP: 1673 (100)	BP: 993 (59.3)	BP: n.a.	BP: n.a.
Kyto et al. [31] (2019/Finland/ROS)	Total: n.a.	Total: n.a.	Total: n.a.	Total: n.a.
	MP: n.a.	MP: n.a.	MP: n.a.	MP: n.a.
	BP: n.a.	BP: n.a.	BP: n.a.	BP: n.a.
Rubino et al. [32] (2021/Italy/ROS)	Total: 395 (100)	Total: n.a.	Total: 59 (14.9)	Total: n.a.
	MP: 314 (100)	MP: n.a.	MP: 46 (14.6)	MP: n.a.
	BP: 81 (100)	BP: n.a.	BP: 13 (16)	BP: n.a.

## Supplementary Materials

The following are available online at https://www.mdpi.com/article/10.3390/jcm10194356/s1, Figure S1: Potential sources of the biases of the studies were analyzed with the use of the components recommended by the ROBINS-I tool.
