# Supplementary figures and images for: Long-Term Outcome of Mechanical and Biological Prostheses in Patients with Left-Side Infective Endocarditis: A Systematic Review and Meta-Analysis
Source: J Clin Med. 2021 Sep 24;10(19):4356. doi: 10.3390/jcm10194356 (PMC8509294; doi:10.3390/jcm10194356)

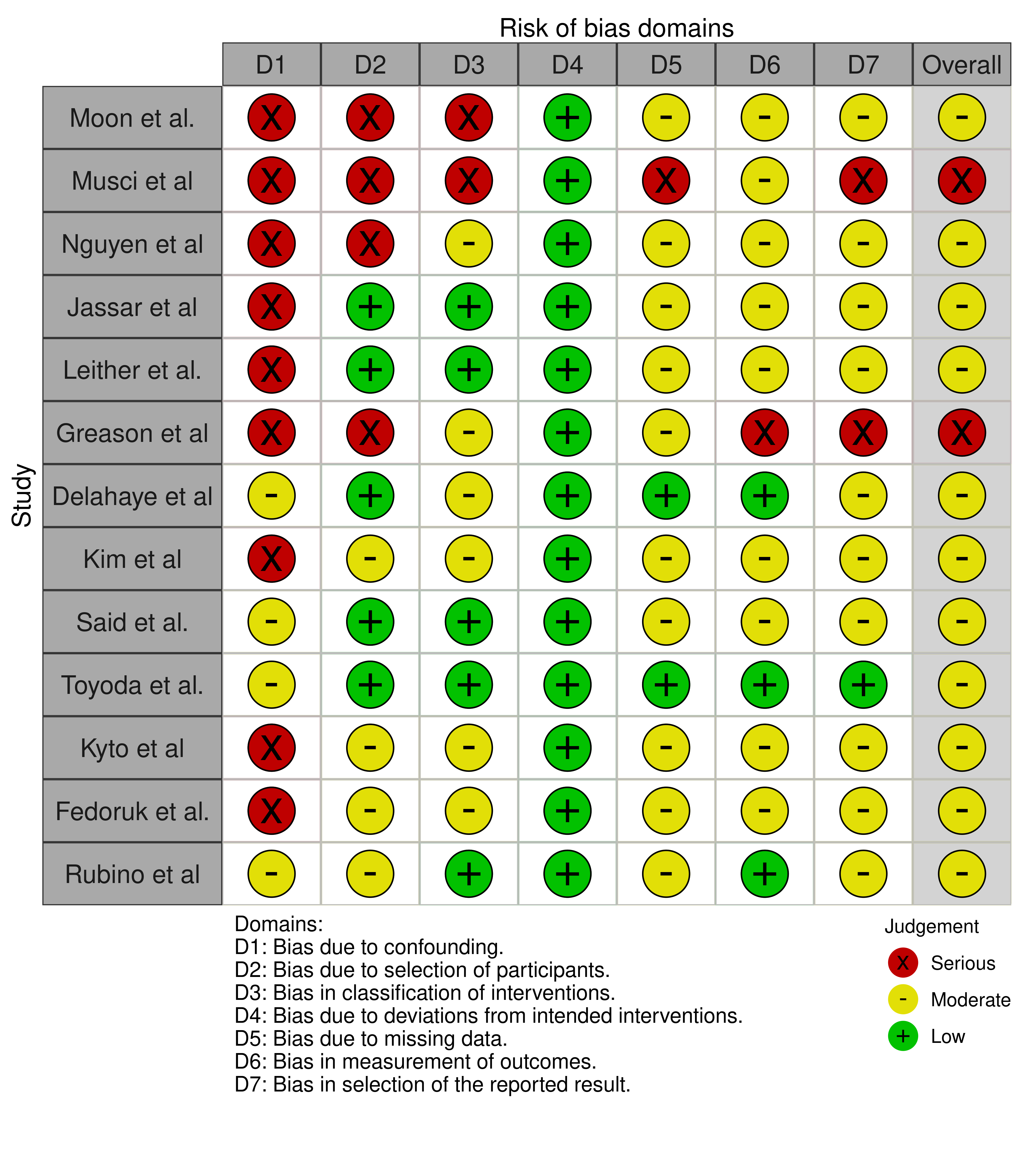

Supplement: Supplementary file 1 [file jcm-10-04356-s001.zip › jcm-1349590-supplementary.png]
